# Inflammation and Cancer: Extra- and Intracellular Determinants of Tumor-Associated Macrophages as Tumor Promoters

**DOI:** 10.1155/2017/9294018

**Published:** 2017-01-18

**Authors:** Gabor J. Szebeni, Csaba Vizler, Klara Kitajka, Laszlo G. Puskas

**Affiliations:** ^1^Avidin Ltd., Alsó Kikötő sor 11/D., Szeged 6726, Hungary; ^2^Synaptogenex Ltd., Őzsuta utca 20995/1, Budapest 1037, Hungary; ^3^Department of Biochemistry, Biological Research Center, Hungarian Academy of Sciences, Temesvári krt. 62, Szeged 6726, Hungary; ^4^Department of Genetics, Biological Research Center, Hungarian Academy of Sciences, Temesvári krt. 62, Szeged 6726, Hungary

## Abstract

One of the hallmarks of cancer-related inflammation is the recruitment of monocyte-macrophage lineage cells to the tumor microenvironment. These tumor infiltrating myeloid cells are educated by the tumor milieu, rich in cancer cells and stroma components, to exert functions such as promotion of tumor growth, immunosuppression, angiogenesis, and cancer cell dissemination. Our review highlights the ontogenetic diversity of tumor-associated macrophages (TAMs) and describes their main phenotypic markers. We cover fundamental molecular players in the tumor microenvironment including extra- (CCL2, CSF-1, CXCL12, IL-4, IL-13, semaphorins, WNT5A, and WNT7B) and intracellular signals. We discuss how these factors converge on intracellular determinants (STAT3, STAT6, STAT1, NF-*κ*B, RORC1, and HIF-1*α*) of cell functions and drive the recruitment and polarization of TAMs. Since microRNAs (miRNAs) modulate macrophage polarization key miRNAs (miR-146a, miR-155, miR-125a, miR-511, and miR-223) are also discussed in the context of the inflammatory myeloid tumor compartment. Accumulating evidence suggests that high TAM infiltration correlates with disease progression and overall poor survival of cancer patients. Identification of molecular targets to develop new therapeutic interventions targeting these harmful tumor infiltrating myeloid cells is emerging nowadays.

## 1. Origin of Tumor-Associated Macrophages

**Figure 1:**
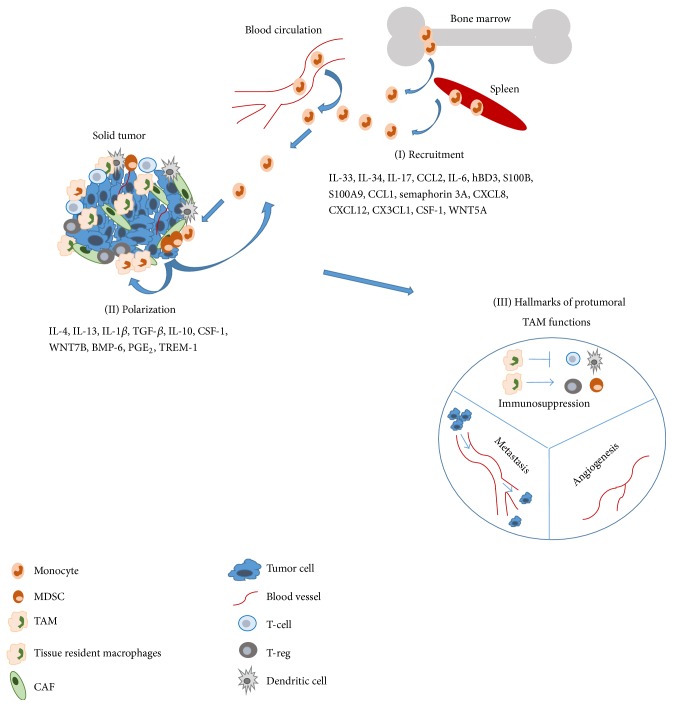
Solid tumors are composed of heterogeneous cell populations, comprising cancer cells, fibroblasts, endothelial cells, pericytes, and leukocytes. Among leukocytes, myeloid cell populations represent a prominent component, in terms of both number and functions, supporting tumor growth and progression. (I) Soluble mediators released by malignant cells or tumor stromal cells recruit different leukocyte populations from the circulation to the tumor site. (II) Infiltrating myeloid cells include immature MDSCs (myeloid-derived suppressor cells) or TAMs. (Factors are listed under (I) and (II) as they appear in the main text.) (III) MDSCs and TAMs exert several protumoral functions such as immunosuppression, angiogenesis, and metastasis. See detailed explanation in the relevant section of the text. CAF: cancer-associated fibroblast; TAM: tumor-associated macrophage; MDSC: myeloid-derived suppressor cell; T-reg: regulatory T-cell.

In 1863 Virchow described the presence of inflammatory leukocytes in tumor tissues and proposed a concept that the “lymphoreticular infiltrate” in the tumor reflects the origin of chronic inflammation at the tumor site [1]. Tumor-associated macrophages (TAMs) represent the major component of the immune infiltrate of the stroma of solid tumors, as TAMs could represent up to 50% of the tumor mass playing a key role in tumor development (Figure 1) [2]. Macrophages are heterogeneous cells with high plasticity representing a wide spectrum of activation states, ranging from the classically activated (M1 macrophages) to the alternatively activated (M2 macrophages) [3]. In cancer, macrophages are a double edged sword as they can exert both anti- and protumoral functions [4]. However until now, bad prognosis associated with high TAM content has been reported in 80% of the tumor cases [5] due to the inhibition of tumor specific immune response and promotion of neoangiogenesis and tumor cell dissemination [4, 6–8]. Identification of molecular targets to develop new therapeutic interventions targeting these harmful tumor infiltrating myeloid cells is emerging nowadays [9]. It has to be remarked that besides TAMs myeloid-derived suppressor cells (MDSCs) represent the major myeloid cells associated with cancer-related inflammation and cancer development. MDSCs based on their cell surface markers have been classified as monocytic (CD11b^+^Ly6C^+^ in mice and HLA-DR^low/−^ CD14^+^CD33^+^ in human) and granulocytic (CD11b^+^Ly6G^+^ in mice and HLA-DR^low/−^ CD15^+^CD33^+^ in human) subsets [7, 10]. Although MDSCs share functional and phenotypic similarities with TAMs the detailed dissection of MDSCs is beyond the scope of this review. MDSCs have recently been reviewed elsewhere [11–13].

Interaction with pattern recognition receptors (which are Toll-like receptors, C-type lectins, retinoic acid inducible gene-like receptors, and nucleotide-binding oligomerization domain-like receptors) leads to the classical proinflammatory and phagocytic M1 activation of macrophages. These receptors recognize pathogen-associated molecular patterns (like LPS), resulting in the activation of innate immunity and the production of proinflammatory cytokines (IL-12, TNF-*α*, etc.). In addition to infections, endogenous molecules released by dying cancer cells, defined as DAMP (damage-associated molecular patterns) (e.g., high-mobility group protein B1, HMGB1, and heat shock proteins, HSPs), can activate the pattern recognition receptors and contribute to set the polarization of macrophages [14]. Moreover, classically activated macrophages are also induced by IFN-*γ*, alone or in concert with microbial stimuli (e.g., LPS), or cytokines (e.g., TNF-*α* and GM-CSF) [3].

Local signals in the tumor microenvironment (IL-4, IL-6, IL-10, PGE_2_, CSF-1, and transforming growth factor-*β*, TGF-*β*) polarize macrophages into alternative, protumoral M2-like cells [7]. In general M2 macrophages execute Th2 responses such as parasite clearance, dampening of inflammation, angiogenesis, and immunosuppression [7]. These protumoral functions endow M2-like TAMs to be the “Trojan horse” in the struggle against cancer (Figure 1).

Recent results have clarified the ontogenetic diversity of macrophages. Hoeffel et al. published that most of the adult tissue resident macrophages arise during fetal liver hematopoiesis from c-Myb^+^ fetal monocytes generated from the late c-Myb^+^ erythromyeloid precursors in the yolk sac [15]. Only microglia originates directly from the yolk sac precursors without monocyte intermediates [15]. Tissue resident macrophages may give rise to TAMs as it is described for microglia in glioma and for Kupffer cells in hepatocellular carcinoma [16]. It is also published that tissue resident macrophages can undergo rapid in situ proliferation under M2 polarizing Th2 cytokine milieu, specially driven by IL-4 [17]. On the other hand TAM progenitors are mainly blood circulating CCR2^+^ monocytes from bone marrow hematopoiesis as it is described in the case of liver, skin, brain, breast, and colon cancer [16]. Cortez-Retamozo et al. showed that TAMs may arise from the spleen, a reservoir of macrophage precursors in K-Ras driven mouse model of non-small cell lung cancer [18], and the same group published that tumor-derived angiotensin II may drive amplification of splenic macrophage progenitors [19]. Under cancer-driven emergency myelopoiesis monocytic MDSCs expand from the bone marrow and from the spleen. These monocytic MDSCs migrate to tumor microenvironment mainly by CCL2 and CSF-1 signaling and may differentiate locally into TAMs [20]. However, it is difficult to ascertain the exact origin of TAMs due to the lack of specific markers distinguishing primitive precursors, tissue resident and tissue-recruited macrophages [7, 16]. Recent review gives an overall dissection about the origin of TAMs from (embryonic or monocytic-derived) tissue resident macrophages and tumor induced, tumor infiltrating (monocytic) macrophages [4].

**Figure 2:**
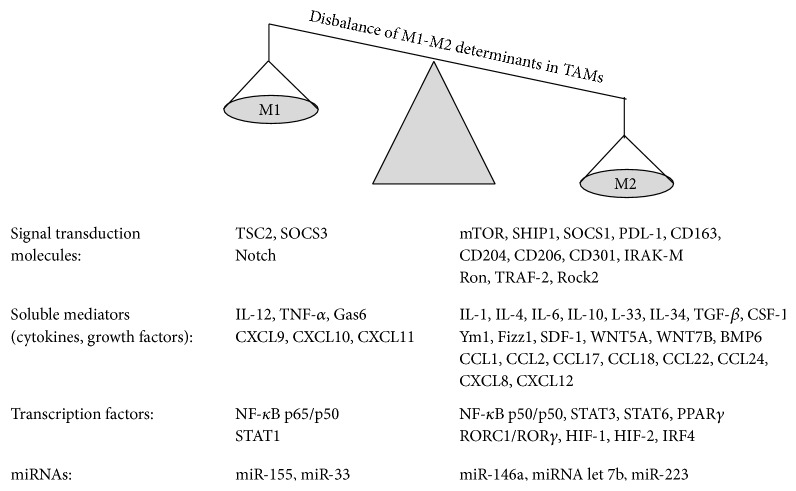
The polarization of TAMs is skewed to alternative activation due to p50/p50 nuclear accumulation, STAT3/STAT6 phosphorylation, and RORC1 transcription factor activation. M1 polarization promotes the expression of proinflammatory cytokines (IL-12, TNF-*α*) and chemokines (CXCL9, CXCL10, and CXCL11) whereas TAMs show anti-inflammatory phenotype (IL10, TGF-*β*) and the expression of CCL2, CCL17, CCL18, CCL22, and CCL24. M1-like (miRNA155, miR-33) and M2-like (miRNA146a, miRNA let 7b, and miR-223) miRNA profile are also a characteristic of macrophage polarization. See detailed explanation in the relevant section of the text.

Molecular profiling studies revealed the expression pattern of TAMs as M2-like macrophages (Figure 2). TAMs produce proangiogenic growth factors: VEGF-A, VEGF-C, EGF, and TGF-*β*, metastatic enzymes: matrix metalloproteinases (MMP2, MMP9), cysteine cathepsin proteases [7, 8], immunosuppressive factors: Arginase I (ArgI), which withdraws the substrate L-arginin from inducible nitric oxide synthase (iNOS), high IL-10 and TGF-*β* levels, and chemokines, such as CCL2, CCL17, CCL18, CCL22, and CCL24 [8]. Moreover, alternatively activated macrophages show high YM1, FIZZ1 expression, Dectin-1^high^, MGL^high^, and scavenger receptor^high^ profile [3, 8]. M2 macrophages express the hemoglobin scavenger receptor CD163, mannose receptor MRC1/CD206, macrophage scavenger receptor I (CD204), and macrophage galactose C-type lectin 1 (MGL1/CD301) [14, 21] as well as low levels of MHC II [22]. Nevertheless it has to be remarked that TAMs from murine fibrosarcoma model upon LPS treatment showed also typical M1 IFN-inducible cytokine expression: CCL5 (also known as RANTES), CXCL9, CXCL10, and CXCL16 due to active IRF3-dependent pathway [23]. Importantly, different tumor types or different tumor stages and tumor tissue regions are characterized by the presence of heterogeneous macrophage populations [5]. It has to be mentioned that in experimental systems it is advised and essential to define the polarization signal since different transcriptome profiles are associated with different stimuli in the M1-M2 transitions [24].

An indirect proof for the decisive role of M2 type macrophage polarization in tumor pathogenesis is provided by the antitumor effect of bacterial infections observed in diverse models. Infections seem to underlie many of the documented cases of melanoma regression [25]. The phenomenon might be analogous to the well-known antitumor effect of Coley's toxins and other related immunomodulating strategies [26]. Interestingly, vaccination with type 1 immunity-driving pathogens was shown to provide life-long protection against melanoma by numerous large-scale clinical studies [27, 28]. The most plausible explanation is pathogen-induced alteration of macrophage polarization, as we demonstrated in our mouse model based on a case study of spontaneous melanoma regression [29].

## 2. Extracellular Signals Driving the Recruitment and Functions of Tumor-Associated Macrophages

Macrophages are educated by the cytokine-chemokine milieu in the tumor microenvironment where TAMs have been shown generally but not exclusively to acquire the hallmarks of alternatively activated M2 macrophages (Figure 2), associated with inhibition of inflammation and immune responses, angiogenesis, and tissue remodeling [3]. We review here the main extracellular signals which recruit and polarize M2 or M2-like tumor-associated macrophages (Figure 1).

Certain cytokines, IL-4 and IL-13, are strong inducers of an alternative (M2) form of macrophage activation [3, 7]. In addition, IL-33, a nuclear alarmin, a cytokine of the IL-1 family, is associated with Th2 and M2 polarization [30]. Another newly discovered cytokine IL-34 expressed by osteosarcoma cells sharing CSF-1R with CSF-1 recruited M2 macrophages, and its overexpression increased tumor growth, vascularization, and metastasis [31]. Macrophages can also be polarized into an “M2-like” state, which shares some but not all the signature features of M2 cells. In this regard, antibody immune complexes together with LPS or IL-1, glucocorticoids, TGF-*β*, and IL-10 result in M2-like functional phenotypes that share properties with IL-4- or IL-13-activated macrophages [32]. Among extracellular factors, autocrine IL-10 production also regulates TAM functions inhibiting M1 polarization through suppression of IL-12 [33]. The tumor promoting phenotype of CD14^+^ blood monocytes and TAMs was IL-1*β*-IL1R axis dependent in human renal cell carcinoma patients [34]. Myeloid compartment specific deficiency in TGF-*β* receptor II showed lower susceptibility to DSS induced colitis-associated cancer along with lower percentage of F4/80^+^ TAM infiltration and downregulation of STAT3 phosphorylation in the colonic adenoma tissue [35]. Further reinforce the role of TGF-*β* in cancer-related inflammation that TGF-*β* not only recruits and polarizes M2 macrophages but also regulates their MMPs expression, which in a mutual response release more TGF-*β* from extracellular matrix store [36]. TGF-*β* plays an important role in the regulation of inflammation since TGF-*β* might contribute to Th17 differentiation and the release of IL-17 [37]. Interleukin-17 has been shown to play a role in the recruitment of IL17RA and IL17RC expressing TAMs in lung cancer [38].

A series of chemoattractants, chemokines, orchestrate the recruitment of monocytes/macrophages; the chemokine CCL2 (also known as MCP-1) was first discovered as a tumor-derived chemotactic factor which recruits these cells to tumor tissues [39]. Several studies reported the pivotal role of tumor cell- or tumor-associated stroma-derived CCL2, as master regulator of monocyte/macrophage recruitment to the tumor site [7]. Along with IL-6, CCL2 also promotes survival and differentiation of myeloid monocytes [40]. In oral carcinoma the tumor cell-produced *β*-defensin-3 (hBD3, which was discovered as an antimicrobial peptide) has been associated with CCR2 dependent TAM trafficking [41]. The Ca^2+^ binding S100 proteins, released from, for example, necrotic cells of the tumor mass, serve as DAMPs, alarmin molecules to the immune system; one member, the S100B molecule, promoted glioma growth by TAM chemoattraction through CCL2 upregulation and induction of STAT3 [42]. Another Ca^2+^ binding S100 protein, S100A9, level correlated with high CD11b^+^ myeloid cell infiltration and increased tumor growth in murine breast cancer model [43]. Eruslanov et al. showed elevated CCL1 expression both in human tumor cells and in CD11b^+^CCR8^+^ myeloid cells of human bladder and renal cell carcinomas. The circulating monocytic CD33^high^CD11b^+^ and granulocytic CD33^low^CD11b^+^ myeloid cell subset as well as CD11b^+^ tumor infiltrating TAMs showed increased expression of the CCL1 chemokine receptor CCR8. The tumor infiltrating CD11b^+^CCR8^+^ myeloid subset produced significant amount of proinflammatory IL-6 and angiogenic VEGF and induced the differentiation of CD4^+^FoxP3^+^ regulatory T-cells [44]. Semaphorins were discovered as axonal growth cone guidance molecules; semaphorin 3B induced CXCL8 (IL-8) production by tumor cells in a Neuropilin-1 dependent manner, which recruited TAMs fostering a prometastatic environment surprisingly with tumor reduction [45]. Hypoxia induced another member of the group, semaphorin 3A, which attracted TAMs via PlexinA1/PlexinA4 and Neuropilin-1 holoreceptor followed by VEGFR1 activation leading to immunosuppression and angiogenesis. Deletion of Neuropilin-1 favored TAM localization in normoxic tumor regions hampering their angiogenic and immunosuppressive functions, thereby impeding tumor growth and metastasis [46]. Stromal cell-derived factor-1 (SDF-1), also known as CXCL12, drives TAM accumulation and survival in hypoxic areas of tumors [47]. CXCL12 also enhances the scavenger receptor CD163 and VEGF expression, shaping monocyte differentiation toward proangiogenic and immunosuppressive phenotype [48]. Fractalkine, another chemokine, CX3CL1, has also been implicated in the recruitment of macrophages since the administration of anti-CX3CR1 antibody decreased macrophage infiltration and angiogenesis in breast cancer [49].

Tumor cells or tumor-associated stromal cells produce hematopoietic growth factors (CSF-1, GM-CSF, and IL-34) which increase the expansion of the monocyte-macrophage lineage [5]. Inhibition of colony-stimulating factor-1 (CSF-1, also known as M-CSF), the primary regulator of tissue macrophage production, or the blockade of CSF-1 receptor (also known as CD115 or c-fms) led to decreased macrophage infiltration and less mammary tumor growth [50]. Depletion of CD115^+^ F4/80^+^ macrophages during early hyperplastic stage with anti-CSF-1R antibody (M279) reduced primary tumor growth and lung metastasis [51]. CSF-1 influences also the maturation of tumor-associated macrophages; in CSF-1 deficient animals macrophages remained more immature with lower expression of IL-1*β*, TNF-*α* [52]. Small interfering RNA against CSF-1 caused decreased neuroblastoma tumor growth with less macrophage infiltration, lower MMP12 level, and angiogenesis [53]. It has been published that STAT1 binds to Csf1 promoter in tumor cells; by this way STAT1 could regulate breast tumor-derived CSF-1 production which drives to the recruitment, in situ proliferation, and M2 phenotype of CD11b^low^ F4/80^high^ TAMs [54].

Not only cytokines, chemokines, and growth factors but also other types of molecules, signal transducers, could influence or determine the M2/M2-like polarization of TAMs. The Wnt gene family consists of lipid modified signaling glycoproteins; the WNT5A expression in human breast tumors correlated with the number of CD163^+^ TAMs, along with induction of immunosuppressive IL-10 and TGF-*β* [55]. Another Wnt family member WNT7B showed colocalization with CD68^+^/CD163^+^ TAMs. Tumor growth in this MMTV-PymT-driven mouse breast cancer was dependent on the WNT7B expression by myeloid cells since in the CSF-1R promoter dependent conditional knock-out model the myeloid selective ablation of Wnt7b reduced tumor growth beyond the failure of the angiogenic switch [56]. In renal cell carcinoma patients, the triple positivity for bone morphogenetic protein-6 (BMP-6, a member of the TGF-*β* superfamily) and CD68 as well as IL-10 positivity in biopsies was correlated with worse cumulative survival, indicating that BMP-6 induces M2 macrophage polarization with IL-10 production via activation of Smad5 and STAT3 [57]. The expression of TREM-1 (triggering receptor expressed on myeloid cells 1, a receptor belonging to the immunoglobulin superfamily) mainly on monocytes/macrophages has been implicated in the severity of chronic inflammation and malignancies [58]. Yuan et al. proposed a mechanism in which tumor cell-derived cyclooxygenase-2 (Cox-2) dependent PGE_2_ induces TREM-1 expression in CD68^+^ TAMs via EP1/EP4 receptors [59]. The same group has also published that TREM-1 prolongs the survival of macrophages through the induction of the antiapoptotic protein Bcl-2 [60]. Both tumor and tumor stroma-derived Cox-2 are associated with poor prognosis. Chen et al. described that myeloid specific ablation of Cox-2 reduced tumor burden and TAM infiltration. Moreover Cox-2 deficiency lowered the M2 immunosuppressive profile of TAMs and enhanced CD8^+^ T-cell immunosurveillance [61].

## 3. Intracellular Determinants of the Recruitment and Functions of Tumor-Associated Macrophages

The inflammatory milieu of the tumor microenvironment affects different intracellular master regulators of TAMs such as Signal Transducer and Activator of Transcription factors (STAT3 and STAT6 for M2, STAT1 for M1 polarization), the nuclear factor-*κ*B (NF-*κ*B), RORC1, and HIF-1. Classical M1 and alternative M2 polarizing determinants are outlined in Figure 2.

Tumor infiltrating myeloid cells undergo a functional change in response to STAT3 activation. This event promotes angiogenesis through VEGF and bFGF induction [62]. STAT3 activation is a key determinant establishing tolerance during immune escape, both in adaptive and in innate effector cells [63]. A pioneer study by Cheng et al. reported that disruption of STAT3 signaling in macrophages or in bone marrow-derived dendritic cells restored the responsiveness of tolerant T-cells from tumor bearing mice [64]. The same group published that the ablation of STAT3 in the hematopoietic compartment combined with CpG treatment resulted in the rapid activation of the innate immunity (high IL-12, TNF-*α*) which forced T-cell response leading to regression of preestablished B16 melanoma tumors [65]. Recently the same group further explored the role of STAT3 in myeloid cells establishing cancer favoring microenvironment. T-cells exert immunosurveillance and constrain myeloid cell accumulation in premetastatic tissue, but this is resisted by STAT3 in myeloid cells. Ablation of STAT3 in myeloid cells led to higher T-cell activation, higher IFN-*γ*, granzyme B production, and consequently myeloid cell apoptosis in the premetastatic niche [6]. The inhibition of STAT3 phosphorylation by oleanolic and corosolic acids, two naturally occurring triterpenes, suppressed tumor-mediated M2 polarization and IL-10 production by macrophages [66]. Other dietary compounds, including n-3 polyunsaturated fatty acids (PUFAs), which have been described as anti-inflammatory and anticancer agents [67, 68], have been recently shown to alter M2 macrophage polarization during the inflammatory response in colon tissue by targeting the epigenetic regulation of gene expression [69]. This novel finding might open new therapeutic potential for dietary PUFAs; however more studies are needed to clarify their potential role in modulating TAMs in cancer. Lovastatin, a cholesterol biosynthesis inhibitor, enhanced tumor infiltration by effector T-cells and reduced the number of immunosuppressive and proangiogenic M2-like TAMs [70]. According to our previous results, where we showed that PUFAs can modulate cholesterol induced inflammatory gene expression [71], we speculate that lipid homeostasis in the tumor microenvironment might have direct or indirect role in macrophage polarization and in local immune functions.

STAT6 is also associated with M2 polarization and STAT6^−/−^ mice showed less tumor growth and metastasis due to enhanced CD8+ Th1 response [72]. The same group reported that survival of tumor bearing STAT6^−/−^ mice and reduction in metastasis are dependent on the restored capacity of TAMs to produce tumoricidal NO [73]. STAT6, in cooperation with Krüppel-like faktor-4 (KLF-4), induces a series of genes responsible for skewing macrophages toward M2, for example, mannose receptor (CD206), Ym1, Fizz1, ArgI, PPAR-*γ* [74], and inhibits M1 genes like TNF-*α*, CCL5, and iNOS [32].

Whereas STAT3 and STAT6 activation occurs in response to M2 signals (IL-10 or IL-4, resp.), STAT1 is activated by M1 polarizing signals (LPS or IFN-*γ*) [75]. IFN-*γ* triggers JAK-mediated tyrosine phosphorylation and dimerization of STAT1 which is fundamental for its DNA binding to various M1 promoter genes (e.g., iNOS, MHCII, and IL-12) [74]. STAT1 also mediates the expression of SOCS3 which inhibits STAT3 activation [76].

In the tumor microenvironment release of danger-associated molecular patterns (e.g., HMGB1, HSPs), IL-1*β* and TNF-*α* lead to NF-*κ*B activation and translocation of the p65/p50 heterodimer to the nucleus which favors M1 polarization of macrophages although accumulation of the p50 inhibitory homodimer mediates defective responsiveness of TAMs and M2 polarization, associated with inhibition of proinflammatory mediators [77, 78]. TAMs from NF-*κ*B p50^−/−^ tumor bearing mice showed M1 profile (IL-12^high^/IL-10^low^), paralleled by increase in vivo production of the Th1 cytokines (IFN-*γ*) by splenocytes and delayed tumor growth [77]. Activation of NF-*κ*B is controlled by I*κ*B kinase *β* (IKK*β*). However, IKK*β* activity is also required to inhibit the M1 or “classical” macrophage activation, resulting in inhibition of STAT1-driven gene expression and downmodulation of IL-12, MHCII, and iNOS expression [79]. The deletion of IKK*β* restored TAM responsiveness which was manifested in tumor growth inhibition, low IL-10, low ArgI, and high IL-12 production [80]. Negative regulators of TLR signaling or NF-*κ*B activation (SIGIRR/TIR8, SHIP1, and SOCS1) have been shown to contribute to the tolerant phenotype of macrophages [81]. TGF-*β* dependent induction of IRAK-M, an inactive serine/threonine kinase, was associated with the M2 phenotype of TAM in a mouse model of lung cancer [82]. Interestingly, IRAK-M expression was paralleled by altered epigenetic regulation, where ERK1/2 activation in a MyD88-independent way caused histone modifications at the IL-10 promoter gene, activating its transcription [83].

The generation of TAMs relies on the persistent stimulation by signals of the chronic inflammatory tumor microenvironment. We recently identified retinoic-acid-related orphan receptor (RORC1/ROR*γ*) as a key driver of the differentiation of TAMs and MDSCs during “emergency” granulomonocytopoiesis in cancer. RORC1 suppresses negative regulators (SOCS3 and Bcl3) and promotes not only positive regulators of granulomonocytopoiesis (CEBP*β*, PU.1) but also the maturation of macrophages (IRF8). RORC1 facilitates the accumulation of immunosuppressive immature MDSCs, prolongs their survival, and polarizes TAMs to M2-like tumor promoting phenotype [10].

Hypoxia (low oxygen tension) is a hallmark feature of almost all solid tumors, often characterized by high infiltration of macrophages, contributing to angiogenesis [84], cytotoxic T-lymphocyte suppression, and tumor progression [85, 86]. Hypoxia stabilizes HIF-1*α*, a prototypical member of the family of hypoxia-inducible transcription factors, by preventing its ubiquitination and subsequent proteasomal degradation. This event results in dimerization of HIF-1*α* with the HIF-1*β* subunit and its nuclear translocation to activate the transcription of genes involved in the cellular adaptation to low oxygen supply. Hypoxic tumor microenvironment also has a fundamental effect on myeloid cell infiltrate which is linked to HIF-1 and HIF-2 factors (HIFs) [84]. Group of Semenza delineated one mechanism in the establishment of hypoxia (HIFs) induced deleterious tumor stroma organization. Hypoxia induces CCL5 expression in mesenchymal stem cells which binds also to hypoxia induced CCR5 in breast cancer cells leading to higher tumoral CSF-1 expression which drives the recruitment of TAMs and MDSCs. The recruitment of these myeloid cells promotes breast tumor growth and metastasis [87]. While hypoxia activates NF-*κ*B through the activation of IKK*β* [88], NF-*κ*B is also a critical transcriptional activator of HIF-1*α* in macrophages [89]. It was reported that HIF-2*α* deficient TAMs expressed lower levels of the chemokine receptor CXCR4 and failed to accelerate tumor growth [90]. Accumulating evidence suggests that tumor infiltrating myeloid cells support tumor development, wherein hypoxia can promote myeloid cell mobilization and homing to tumors through the CXCL12-CXCR4 axis. In the local hypoxic areas of the tumor microenvironment HIF-1*α* triggers CXCL12 (SDF-1) expression which in turn increases migration, adhesion, and homing of CXCR4^+^ progenitors [91]. As a positive regulatory loop hypoxia further increases the chemotactic responsiveness of monocytes/macrophages through the HIF-1*α*-mediated upregulation of CXCR4 [92]. Moreover, hypoxia can mediate MDSC expansion and recruitment from the spleen to the tumor microenvironment and convert them from specific to nonspecific suppressors, promoting their differentiation to TAMs [85]. It has been reported that hypoxia also augments macrophage-mediated T-cell suppression, as targeted deletion of HIF1-*α* in macrophages inhibited ArgI production and T-cell suppression, resulting in tumor growth inhibition [86]. The immune regulator, programmed death-1 ligand-1 (PD-L1), has been identified as a HIF-1*α* dependent specific factor and potential therapeutic target upregulated by hypoxia in splenic MDSCs and TAMs in murine lung, breast, and colon cancer and melanoma [93]. Laoui et al. showed that tumor infiltrating Ly6C^hi^ monocytes give rise to both MHCII^hi^ and MHCII^low^ CD11b^+^F4/80^+^ macrophages in Lewis lung carcinoma (LLC) model. The latter localized mainly in hypoxic regions with higher expression of typical M2 markers. The prolyl-hydroxylase domain 2 haplodeficiency with the phenotype of vessel normalization did not alter M1/M2 TAM distribution rather diminished the expression of hypoxia regulated M2 associated genes and proangiogenic activity of MHCII^low^ CD11b^+^F4/80^+^ macrophages [94]. HIF-1 has been identified to link the inflammatory and oncogenic pathways, as it was shown that IL-1*β* upregulated HIF-1*α* protein through a classical inflammatory signaling pathway involving NF-kB and COX-2 [95].

New molecular players have been identified in TAM polarization as potential candidates or targets of therapeutic interventions. It has been described that the TSC2-mTOR pathway is a key determinant in the differentiation of monocytes to M2 angiogenic macrophages, and the mTOR inhibitor rapamycin caused M1 polarization. On the contrary when mTOR was active, knocking-down the intrinsic mTOR inhibitor TSC2, TAMs produced more IL-10 and both tumor growth and angiogenesis increased [96]. Forced signaling of Notch increased M1 macrophages with IL-12 production, whereas compromised Notch signaling caused M2 polarization of macrophages favoring B16 melanoma and LLC lung carcinoma tumor growth [97]. Among tumor infiltrating leukocytes TAMs produced the mitogen Gas6 accelerating primary tumor growth and metastasis [98]. During alternative activation of macrophages IL-4 induced c-myc upregulated STAT6 and PPAR-*γ*, thus contributing to the protumoral phenotype of TAM [99]. Specific ablation of Ron receptor tyrosine kinase in the myeloid compartment inhibited prostate cancer growth which was restored by the depletion of CD8^+^ but not CD4^+^ T-cells [100]. TNF receptor-associated factor 2 (TRAF-2) suppresses the proinflammatory cytokine production in macrophages. Recently it has been published that TRAF-2 controls the fate of IRF5 and c-Rel by proteasomal degradation via TRAF3 and cIAP. Myeloid cell specific deletion of TRAF-2 resulted in less tumor growth and lower TAM infiltration along with higher intratumoral IFN-*γ* produced by higher percentage of CD4^+^ and CD8^+^ T-cell infiltration [101].

## 4. Posttranscriptional Regulation and Role of MicroRNAs in the Polarization of Tumor-Associated Macrophages

Nowadays, the regulatory role of microRNAs (miRNAs) that control both physiological and pathological processes is under intensive investigation. The last decade has seen an explosion of knowledge about miRNAs in cancer, hematopoiesis, and inflammation. As mutations of oncogenes or tumor suppressors, deregulation of miRNAs can drive oncogenesis and tumor progression. The nonneoplastic cells in the tumor stroma, as TAMs, also show an altered miRNAs expression. Although miRNAs regulate posttranscriptional gene expression intracellularly these regulators are not considered only intracellular determinants of the polarization of macrophages in this review since intercellular communication by miRNA transporting exosomes has been described not only between malignant cells and TAMs but also among the variety of cellular components of the inflammatory microenvironment [102–104]. A complete overview of miRNAs in terms of cancer-related inflammation is beyond the scope of this review; we summarize the recent results on the effects of key miRNAs on monocyte/macrophage lineage development and on the polarization of TAMs. The detailed summary of miRNAs in relation to macrophage polarization and immune response is recently reviewed elsewhere [105, 106].

The miR-146 family includes two members: miR-146a and miR-146b. Although their sequences differ only in two nucleotides, they are encoded on different chromosomes [107]. In 2006 Taganov and his colleagues showed the NF-*κ*B dependent upregulation of the endotoxin responsive (TLR2-, TLR4-, and TLR5-dependent) miR-146a in the THP-1 human monocytic cell line. They identified interleukin-1 receptor-associated kinase 1 (IRAK1) and TNF receptor-associated factor 6 (TRAF6) adaptor molecules as the main targets of miR-146a in the TLR pathway [108]. MiR-146a represents a negative feedback loop dampening the signals upstream NF-*κ*B, with relevance in endotoxin induced tolerance [109]. MiR-146a promotes the binding of the transcriptional repressor Relb to the TNF-*α* promoter, leading to the inhibition of TNF-*α* expression. In addition, miR-146a also promotes RBM4-Ago2 interaction, with the assembly of the miRNA-induced silencing complex which disrupts translation of TNF-*α* in THP-1 cells [110]. It has been published in a* Listeria monocytogenes* infection model that miR-146a controls the expansion and recruitment of inflammatory monocyte precursors by targeting Relb and CCR2 in the Ly6C^high^ subset [111]. Induced expression of miR-146a in glioma cells has been reported upon DHA treatment and irradiation with 10 Gy [112]. Curtale et al. showed that the anti-inflammatory activity of IL-10 at least partly relies on the induction of miR-146b on a STAT3 dependent manner; miR-146b modulates TLR4 signaling pathway by dampening MyD88, IRAK1, and TRAF6 [113].

In two different comparative studies, performed with bone marrow-derived macrophages (BMDMs), M1 polarization by LPS/IFN-*γ* induced miR-155, while M2 polarization by IL-4 induced miR-146a [114, 115]. miR-155 is also upregulated in macrophages by NF-*κ*B activation, via stimulation of TLR2, TLR4, TLR5, and TLR9 [116]. Interestingly, miR-155 targets negative regulators of inflammation: suppressor of cytokine signaling 1 (SOCS1) [117], B-cell lymphoma-6 protein (BCL6) [118], and Src homology-2 domain-containing inositol 5-phosphatase 1 (SHIP1) [119]. Moreover, miR-155 silences IL-13 receptor (IL13RA) which promotes M2 polarization of macrophages [120], stabilizes the TNF-*α* transcript, by increasing its half-life [121], and targets CEBP/*β*, which is an M2 associated transcription factor with increased activity in TAMs [122, 123]. MiR-155^−/−^ mice showed increased tumor growth [124] and miR-155 in vivo knockdown in the myeloid compartment impaired the ability of these cells to mount a proinflammatory response, which also increased tumor growth [125]. In accordance with the aforementioned observations, miR-155 overexpressing TAMs were repolarized from M2 to M1 ex vivo [115]. In chronic inflammation elevated levels of miR-155 downregulate mismatch repair proteins [126] and block the cell-cycle regulatory WEE1 kinase [127]; this promutagenic activity of miR-155 further forces the link between inflammation and cancer.

The experimental results about the role of miRNAs in the polarization of myeloid cells such as macrophages are often controversial due to the different cell types, stimuli, and tumor models used in the studies of different laboratories [106]. In human metastatic breast cancer patients the CSF1-R^+^ myeloid cells showed CSF1-ETS2 induced higher expression of miR-21 and miR-29a which correlated with higher metastatic tumor burden [128]. However, Wang et al. reported that miR-21 as an endogenous brake prevents PGE_2_ mediated M2 polarization via targeting STAT3 in murine peritoneal macrophages [129]. The passenger strand of miR-511 is the bioactive strand (miR-511-3p) which is encoded within the fifth intron of MRC1 (macrophage mannose receptor, CD206) gene. Hence, during alternative (M2-like) activation of macrophages MRC1 expression is accompanied by miR-511-3p synthesis. Surprisingly, the overexpression of miR-511-3p in hematopoietic cells inhibited LLC tumor growth, disrupted vessel morphology, and as a negative regulator tuned down protumoral genes such as matrix remodeling enzymes, scavenger receptors, and TGF-*β*. Squadrito et al. showed that miR-511-3p targets Rho-associated coiled-coil containing protein kinase 2 (Rock2), a serine-threonine kinase that phosphorylates IRF4, a transcription factor that promotes M2 activation of macrophages [130]. In this way miR-511 serves to establish a threshold, as an endogenous molecular “brake” to suppress the M2 polarization of TAMs. Chaudhuri et al. reported that overexpression of miR-125b mediated M1 polarization and increased the response of macrophages to IFN-*γ* which resulted in higher MHCII, CD40, CD80, and CD86 expression; moreover miR-125b overexpressing RAW264.7 macrophages were more effective killers of EL4 thymoma tumor cell line [131]. Banerjee et al. differentiated murine bone marrow cells by GM-CSF (GM-BMM) and CSF-1 (M-BMM) to establish macrophage cultures. TLR2 and TLR4 ligands upregulated miR-125a-5p on MyD88 dependent manner. Overexpression of miR-125a-5p in GM-CSF differentiated macrophages reduced M1 cytokine production (IL-12, TNF-*α*, and iNOS) and increased Arg1, characteristic of M2 phenotype, upon IL-4 treatment. Surprisingly miR-125a-5p overexpression diminished the bactericidal activity in contrast increased phagocytic activity of GM-BMM cells [132]. Forced activation of Notch repressed tumor growth and subverted TAM phenotype to IL-12^high^ and iNOS^high^; furthermore active Notch signaling downstream upregulated miR-125a in bone marrow-derived macrophages (BMDM) mainly by LPS+IFN-*γ* stimuli. MiR-125a mimetics promoted IL-12, iNOS, and TNF-*α* expression in BMDMs and restored T-cell proliferation. Finally, Zhao et al. concluded that miR-125a targets the suppressor of HIF-1*α*, FIH1, and IRF4 to promote M1 and suppresses M2 polarization of macrophages. [133]. The inconsistency about the M1 or M2 promoting role of miRNA125a may arise from the different source of cells and stimuli used by different laboratories so further studies should clarify this discrepancy. Another miRNA, miRNA let-7b, has been shown to be upregulated in prostatic TAMs (established by the incubation of human blood monocytes with PCa prostate cancer cell conditioned medium) and modulates IL-12, IL-23, IL-10, and TNF-*α* and promotes mobility of PCa cells and proangiogenesis of PCa-conditioned TAMs [134]. Inhibition or macrophage specific deletion of miR-33, a miRNA playing role in cellular metabolism, increased oxidative phosphorylation and induced M2 polarization associated gene profile (ArgI^high^, Mrc1^high^); additionally miR-33 deficient macrophages promoted the differentiation of T regulatory cells. MiR-33 regulates macrophage inflammation via targeting of AMP-activated protein kinase thereby promoting glycolysis and M1 polarization [135].

MiR-223 has been described as a novel regulator of macrophage polarization targeting Pknox1 transcription factor; miR-223 limits proinflammatory activation of macrophages and enhances the alternative anti-inflammatory responses [136].

## 5. Conclusions

In conclusion, accumulating evidence suggests that further identification of novel molecules and mechanisms involved in macrophage recruitment and/or polarization offers the basis for novel therapeutic approaches against harmful myeloid cells educated by the developing tumors. Reeducating TAMs, tuning the balance from M2 to M1 in the tumor microenvironment might be a holy grail of macrophage-targeted therapies.
